# Supplementary material for: Maternal Exposure to Potentially Toxic Metals and Birth Weight: Preliminary Results from the DSAN-12M Birth Cohort in the Recôncavo Baiano, Brazil
Source: Int J Environ Res Public Health. 2023 Jun 23;20(13):6211. doi: 10.3390/ijerph20136211 (PMC10340643; doi:10.3390/ijerph20136211)
Supplement: Supplementary file 1 [file ijerph-20-06211-s001.zip › Supplementary.pdf]

Table S1: Exposure to PTMs in the study population according to deliveries included or not in this analysis.

		FG not collected	FG collected	Total	p-value (X^2)
PbB	< Median	29 (52.7)	30 (48.4)	59 (50.4)	0.639
	≥ Median	26 (47.3)	32 (51.6)	58 (49.6)	
CdB	< 0.6 μg/L	33 (54.1)	35 (53.8)	68 (54)	0.977
	≥ 0.6 μg/L	28 (45.9)	30 (46.2)	58 (46)	
CdB	< 1.0 μg/L	45 (73.8)	53 (81.5)	98 (77.8)	0.295
	≥ 1.0 μg/L	16 (26.2)	12 (18.5)	28 (22.2)	
MnH	< Median	30 (47.6)	37 (54.4)	67 (51.1)	0.437
	≥ Median	33 (52.4)	31 (45.6)	64 (48.9)	
MnTn	< Median	28 (54.9)	25 (46.3)	53 (50.5)	0.378
	≥ Median	23 (45.1)	29 (53.7)	52 (49.5)	
